# The Effect of Geranylgeraniol and Ginger on Satellite Cells Myogenic State in Type 2 Diabetic Rats

**DOI:** 10.3390/cimb46110730

**Published:** 2024-10-31

**Authors:** Nigel C. Jiwan, Casey R. Appell, Raoul Sterling, Chwan-Li Shen, Hui-Ying Luk

**Affiliations:** 1 Department of Kinesiology, Hope College, Holland, MI 49423, USA; jiwan@hope.edu; 2 Department of Kinesiology and Sport Management, Texas Tech University, Lubbock, TX 79406, USA; casey.appell@ttu.edu (C.R.A.); raoul.sterling@ttu.edu (R.S.); 3 Department of Pathology, Texas Tech University Health Sciences Center, Lubbock, TX 79430, USA; leslie.shen@ttuhsc.edu

**Keywords:** high-fat diet, muscle mass, MyoD, myostatin, nutrition, Pax7

## Abstract

Type 2 diabetes (T2D) is associated with increased inflammation and reactive oxygen species (ROS) in muscles, leading to basal satellite cell (SC) myogenic impairment (i.e., reduction in SC pool), which is critical for maintaining skeletal muscle mass. T2D may contribute to muscle atrophy, possibly due to reductions in the SC pool. Geranylgeraniol (GGOH) and ginger can reduce inflammation and enhance SC myogenesis in damaged muscles, thereby alleviating muscle atrophy; however, their effect on basal SC myogenic state and muscle mass in T2D rats is limited. Rats consumed a control diet (CON), high-fat diet with 35 mg/kg of streptozotocin (HFD), a HFD with 800 mg/kg body weight of GGOH (GG), or a HFD with 0.75% ginger root extract (GRE). In the eighth week, their soleus muscles were analyzed for Pax7, MyoD, and MSTN gene and protein expression, SC myogenic state, and muscle cross-sectional area (CSA). The HFD group had a significantly lower number of Pax7^+^/MyoD^−^ and Pax7^+^/MSTN^+^ cells, less Pax7 and MyoD gene expression, and less MyoD and MSTN protein expression, with a smaller CSA than the CON group. Compared to the GG and GRE groups, the HFD group had a significantly lower number of Pax7^+^/MSTN^+^ cells, less MyoD protein expression, and smaller CSA. The GRE group also had a significantly lower number of Pax7^−^/MyoD^+^ and greater MSTN protein expression than the HFD group. Nevertheless, the CON group had a significantly greater number of Pax7^+^/MyoD^−^ than the GG and GRE groups, and a greater number of Pax7^−^/MyoD^+^ cells than the GRE group with a larger CSA than the GG group. GGOH and ginger persevered muscle CSA, possibly through increased MyoD and the ability to maintain the SC pool in T2D rats.

## 1. Introduction

Type 2 diabetes (T2D) is a severe public health concern, and its prevalence could increase to 7.8% of the global population by 2040 [1]. T2D is associated with excessive production of reactive oxygen species (ROS) [2], chronic inflammation [3], and impaired glucose uptake by the skeletal muscles [3]. T2D due to insulin resistance leads to notable changes in skeletal muscles, including muscle atrophy, which is characterized by a reduction in myofiber size and overall muscle mass, ultimately leading to diminished muscle strength [4]. One critical aspect of muscle regeneration involves satellite cells (SCs), which are essential for maintaining muscle mass and facilitating recovery from injury [5].

Quiescent SCs can be activated and then undergo proliferation and differentiation in response to insult (e.g., muscle damage, muscle wasting, etc.) [6,7,8]. SCs are regulated by the sequential up- and downregulation of myogenic transcription factors [6,7,8], namely, the paired box transcription factor-7 (Pax7), myogenic differentiation 1 (MyoD), and myogenin [9]. At rest, quiescent SCs only express Pax7, but upon stimulation (injury, muscle damage), the activation of Pax7^+^ SCs results in the transient upregulation of MyoD, indicating SCs are in the proliferating stage (Pax7^+^/MyoD^+^) [9]. MyoD transcription factor initiates the transcription of myogenin and other muscle-specific genes in differentiating myoblasts into myotubes [10]. In addition to the tightly orchestrated regulation of myogenic transcription factors, the signal for SCs to return to quiescence is critical for maintaining the SC pool and its potency to undergo myogenic progression [11]. However, the mechanism responsible for SCs returning to quiescence is not fully understood. One potential candidate is myostatin (MSTN), which can downregulate MyoD expression during the proliferative stage, resulting in SCs exiting the cell cycle [12] and returning to a quiescent state (Pax7^+^/MSTN^+^) [13,14,15]. Considering the frequent occurrence of myofiber injuries during routine muscle activity and in disease states, continual repair and replenishment of the SC pool are vital for sustaining muscle integrity. Thus, it is important to understand the basal myogenic state of SCs in diabetic muscles, as a decrease in SC pool size could negatively impact skeletal muscle health [16].

Previous studies have consistently shown that diabetic skeletal muscles exhibit impaired SC myogenic progression following muscle injury, particularly affecting SC proliferation and differentiation [17,18,19]. In diabetic rodents, both streptozotocin (STZ)-induced and high-fat diet-induced models have shown reductions in MyoD and myogenin protein expression in the gastrocnemius [17], as well as a decreased number of activated SCs in the plantaris muscle [19] after cardiotoxin injury. Although these studies did not analyze muscle regeneration together with SC function, others have demonstrated that diabetic rodents had smaller regenerating myofibers [20,21]. Taken together, these results suggest that diabetes negatively affects muscle regeneration by impairing satellite cell function. Notably, myostatin, the inhibitor of SC myogenesis and muscle growth, can help keep SCs in a quiescent state [22] and has been shown to be elevated in the circulation of type 1 diabetic patients [23] and in skeletal muscles [24] of T2D patients, whereas other studies have disagreed [25]. Furthermore, impaired SC myogenic progression has been shown to result in muscle atrophy [26], which in turn exacerbates T2D progression (i.e., increased insulin resistance) [27].

Factors contributing to SC impairment in diabetic muscles are multifaceted, including chronic inflammation [28] and high levels of ROS [29]. Therefore, alleviating inflammation and oxidative stress in diabetic muscles is critical for improving the intramuscular milieu, which could potentially improve the SC function. Our previous reports have demonstrated that geranylgeraniol (GGOH), known for its anti-inflammatory properties, and ginger, recognized for its antioxidant properties, have alleviated muscle mass loss in streptozotocin (STZ) and high-fat diet-induced diabetic rats [30,31]. However, it is not known whether SCs contributed to alleviating muscle mass loss. Other studies have shed light on the potential effect of GGOH and ginger on muscle regeneration. Matsubara et al. (2018) reported that when healthy C2C12 cells were incubated with GGOH, there was an increase in myogenin and myosin heavy chain (MHC) protein expression [32]. Similarly, ginger supplementation has shown promising results in improving myotube size and regenerative capacity in healthy and senescent myoblasts [33]. Collectively, these results suggested that GGOH and ginger alone could alter SC myogenic state, which could help prevent muscle mass loss. However, the effect of GGOH and ginger root extract (GRE) supplementation on SC myogenic state in T2D rats has remained unexplored. Therefore, this study aims to investigate the effects of GGOH and ginger on SC myogenic state and muscle cross-sectional area (CSA) in diabetic rats. It was hypothesized that diabetic rats supplemented with GGOH or ginger would preserve muscle CSA and quiescent SC pool.

## 2. Materials and Methods

### 2.1. Animals and Treatments

The details of this ancillary study design were adapted from previously published work [30,31]. Briefly, 40 Sprague-Dawley rats were divided into four groups: regular diet (CON; *n* = 10), high-fat diet (HFD; *n* = 10), geranylgeraniol + high-fat diet (GG; *n* = 10), and ginger root extract + high-fat diet (GRE; *n* = 10). The CON group was fed an AIN-93G diet containing 10% of calories from fat throughout the eight-week study. The HFD group received a high-fat diet consisting of 20% carbohydrates, 22% protein, and 58% fat (catalog #D12492, Research Diets Inc., New Brunswick, NJ, USA) [34]. The GG group was provided the same high-fat diet with 800 mg/kg GGOH (American River Nutrition, LLC., Hadley, MA, USA), which had an 85% purity, requiring 940 mg of GGOH to be incorporated into 1000 g of HFD. This GGOH dosage was selected because previous research has demonstrated that 800 mg/kg improved glucose tolerance, enhanced insulin sensitivity, and reduced proinflammatory adipokines [35]. Lastly, the GRE group was given the high-fat diet supplemented with 0.75% *wt*/*wt* ginger which corresponded to 300 mg/kg body weight (ginger root extract 20% consisting of 18.7% 6-gingerol, 1.81% 8-gingerol, 2.86% 10-gingerol, 3.09% 6-shogoal, 0.39% 8-shogaol, and 0.41% 10-shogaol; Sabinsa Corporation East Windsor, NJ, USA; Lot #G190297) [34]. This dosage for ginger was selected because previous studies have shown that dosages between 100 and 500 mg/kg effectively mitigated diabetic complications in diabetic rat models [36,37,38]. After two weeks on these diets, the HFD, GG, and GRE groups received a 35 mg/kg dose of streptozotocin (STZ) to induce diabetes. Fasting blood sugar levels were measured, and the rats were considered diabetic if fasting blood sugar exceeded 200 mg/dL. Diabetes was confirmed in the HFD, GG, and GRE groups based on these glucose levels. One rat from the GG group, one rat from the HFD group, and three rats from the GRE group were excluded because the rats’ blood glucose levels were below 200 mg/dL. Each rat was individually housed in a cage (temperature: 21 ± 2 °C; a 12 h light–dark cycle). They were provided their assigned diets twice weekly and had unlimited access to food and water. The Texas Tech University Health Sciences Center Institutional Animal Care and Use Committee #19175 approved all conditions and handling of the animals. All experiments were performed according to the relevant guidelines and regulations.

### 2.2. Sample Collection

In the eighth week, blood and soleus muscle were collected from the rats after being fasted for 4 h and stored at −80 °C for further analysis. In addition, the right and left soleus muscles were harvested to perform gene and protein expression and immunohistochemical analysis.

### 2.3. RNA Isolation, RT-qPCR Analysis

A detailed description of muscle tissue homogenization and RNA isolation has been published previously [31]. Muscle samples were analyzed for intramuscular gene expression of *Pax7* and *MyoD* for the CON, HFD, GG, and GRE groups. RNA was isolated from flash-frozen muscle samples (~30 mg) using the RNeasy Fibrous Tissue Mini Kit (Cat. # 74704, Qiagen, Germantown, MD, USA) with the recommended standardized protocol. cDNA was synthesized from 1 μg of total RNA using an iScript reverse transcriptase kit (Bio-Rad, Hercules, CA, USA). Real-time PCR amplification experiments and calculations of relative expression levels were performed following the user manual # 2 ABI PRISM70500 Sequence Detection System (Applied Biosystems, Waltham, MA, USA) with iTaq SYBR Green Supermix (Bio-rad). Pre-designed assays for *Pax7* and *MyoD* were obtained from Integrated DNA Technologies with *β-actin* as the internal control. Duplicate technical replications were performed for each assay, and data were then analyzed using the 2^−ΔΔCt^ method to determine relative fold change in transcript abundance.

### 2.4. Immunohistochemistry

Muscle cross-sections (7 μm) were stained with antibodies against Dystrophin (1:100; Thermofisher, Waltham, MA, USA; Pa5-32388), MHC1 (1:500; Novus, Centennial, CO, USA; NBP2-50298), MyoD (1:100; Proteintech, Rosemont, IL, USA; 18943-1-AP), Myostatin (MSTN; 1:1000; Millipore, St. Louis, MO, USA; AB3239-1), and Pax7 (1:100; Santa Cruz, Dallas, TX, USA; SC-81648). Appropriate secondary antibodies were applied: anti-mouse (1:1000; Thermofisher; R6393) and anti-rabbit (1:1000; Abcam, Waltham, MA, USA; ab7064). Histochemical methods were adapted from previously published methods [39,40]. Briefly, for co-immunofluorescence staining (Dystrophin and Pax7; Dystrophin, MyoD, and Pax7; Dystrophin, MSTN, and Pax7), sections were fixed with ice-cold acetone for 5 min, followed by washing in PBS. Next, sections were blocked with 2% H_2_O_2_ for 10 min, then 1% BSA solution for 20 min. Following blocking, sections were incubated in the primary antibody at 4 °C overnight. After washing, sections were then incubated in the appropriate secondary antibodies. Sections were then re-fixed in ice-cold acetone to prevent migration of the secondary antibodies and re-blocked. The sections were then incubated in the second primary antibody, followed by incubation in the appropriate secondary antibody. Sections were then washed with PBS and 4′,6-diamidino-2-phenylindole (DAPI; Invitrogen, Waltham, MA, USA, D1306) for nuclear staining. The slides were visualized using a Zeiss Axiovert 200m Inverted Fluorescent Motorized Microscope, and images were captured. The number of cells expressing SCs was counted and normalized per 100 fibers. Additionally, muscle CSA was analyzed using Image J (Version 1.54g, National Institute for Health, Bethesda, MD, USA), with 100 muscle fiber areas measured per sample.

### 2.5. Tissue Homogenization and Western Blot Analysis

The details of the Western blot procedure were adapted from previously published work [30,31]. Briefly, frozen muscle samples were homogenized in 10 mL/mg muscle of ice-cold RIPA buffer (Sigma Aldrich, Burlington, MA, USA, 89901) containing 1X protease inhibitor (Sigma Aldrich, PPC1010, 1:100). Homogenate was collected and analyzed for total protein concentration using Pierce™ BCA Protein Assay Kit (Thermofisher, 23225). Aliquots from homogenates were loaded (equal amount of protein) per lane in duplicate and separated by SDS-PAGE. All proteins were run on 10% gels (Bio-Rad, 4561033) for 60 min at 120 V. Samples were then transferred to a polyvinylidene fluoride (PVDF) membrane for immunoblotting via electrophoresis for 150 min at 70 V. The ladder on the membranes was marked with WesternBright ChemiPen (Advansta Inc., San Jose, CA, USA, R-07055-001). Next, non-fat milk powder was used to block the membranes, followed by primary antibodies incubation with Pax7 (1:1000; Santa Cruz; sc-81648), MyoD (1:2000; ProteinTech; 18943-1-AP), myostatin (MSTN; 1:1000; Millipore Sigma, St. Louis, MO, USA; AB 3239-1), and GAPDH (1:4000; Cell Signaling, Danvers, MA, USA; 8884) overnight. Then, membranes were incubated with secondary anti-mouse IgG (1:1000; Cell Signaling; 7076S) and anti-rabbit IgG (1:1000; Cell Signaling; 7074S) for 60 min accordingly. Chemiluminescent substrate (Advanta Inc., K-12043-D10) and the ChemiDoc MP Imaging System (BioRad) were used to picture the stained protein bands. Following imaging, the membranes were stripped with 5X Western Reprobe for 90 min. Band density was analyzed using Image Lab Software 6.1 (BioRad, 12003154). Total protein concentrations were normalized to a housekeeping protein GAPDH and expressed as arbitrary units. See Supplementary Materials for full Western blot images.

### 2.6. Statistical Analyses

Statistical analyses were performed using SPSS (IBM version 29; Armonk, NY, USA: IBM Corp.) for all analyses. Two separate one-way ANOVAs were used to analyze gene expression, protein content, soleus muscle CSA, and satellite cell numbers for the supplemental groups. In addition, Bonferroni post hoc tests were used for pairwise comparisons. The statistical significance was accepted at *p* < 0.05. Data are reported as mean ± SE.

## 3. Results

### 3.1. Satellite Cell Markers

**Figure 1:**
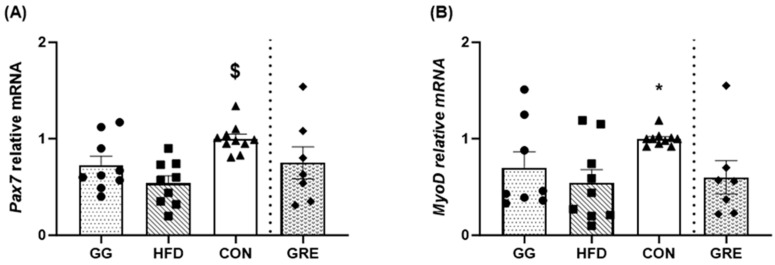
Gene expression analyses [GG: *n* = 9; HFD: *n* = 9; regular diet (CON): *n* = 10; GRE: *n* = 7] for (**A**) Pax7 and (**B**) MyoD. A significant condition effect was observed for Pax7 and MyoD. Two separate one-way ANOVAs were conducted between supplementation groups (GG, HFD, and CON; HFD, CON, and GRE). Values are mean ± SE. $ *p* < 0.05 vs. HFD & GG. * *p* < 0.05 vs. HFD.

**Figure 2:**
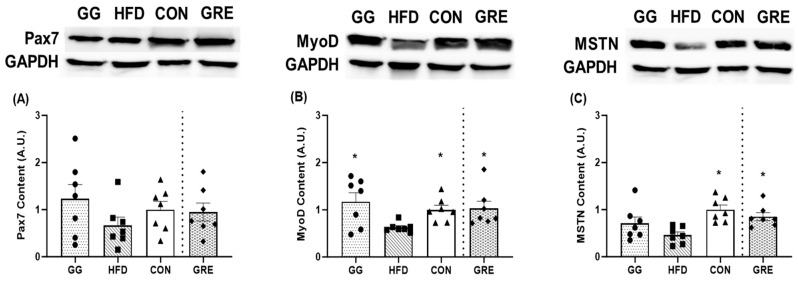
Protein expression analyses for (**A**) Pax7, (**B**) MyoD, and (**C**) MSTN. Protein expression analysis [GG: *n* = 7; HFD: *n* = 7; regular diet (CON): *n* = 7; GRE: *n* = 7] data were normalized to GAPDH. A significant condition effect was observed for MyoD and MSTN. Western blots display an example of protein expression for Pax7, MyoD, and MSTN and corresponding GAPDH in GG, HFD, CON, and GRE groups of rats. Two separate one-way ANOVAs were conducted between supplementation groups (GG, HFD, and CON; HFD, CON, and GRE). Values are mean ± SE. * *p* < 0.05 vs. HFD.

For Pax7 gene expression, CON (1.00 ± 0.05) was significantly greater (*p* < 0.05) than HFD (0.54 ± 0.08) and GG (0.73 ± 0.09) with no differences among all other groups (Figure 1A). For Pax 7 protein content, no significant differences were observed among groups (Figure 2A).

For MyoD gene expression, CON (1.00 ± 0.02) was significantly greater than HFD (0.54 ± 0.14), with no differences among other groups (Figure 1B). For MyoD protein content, GG (2.62 ± 0.43) and CON (2.24 ± 0.21) were significantly greater than HFD (1.41 ± 0.09). Furthermore, GRE (2.30 ± 0.34) and CON (2.24 ± 0.21) were also significantly greater than HFD (1.41 ± 0.09). No differences were observed among other groups (Figure 2B).

For MSTN protein content, CON (0.91 ± 0.09) and GRE (0.78 ± 0.08) were significantly greater than HFD (0.42 ± 0.06), with no differences observed for the remaining groups (Figure 2C).

### 3.2. SC Myogenic State

**Figure 3:**
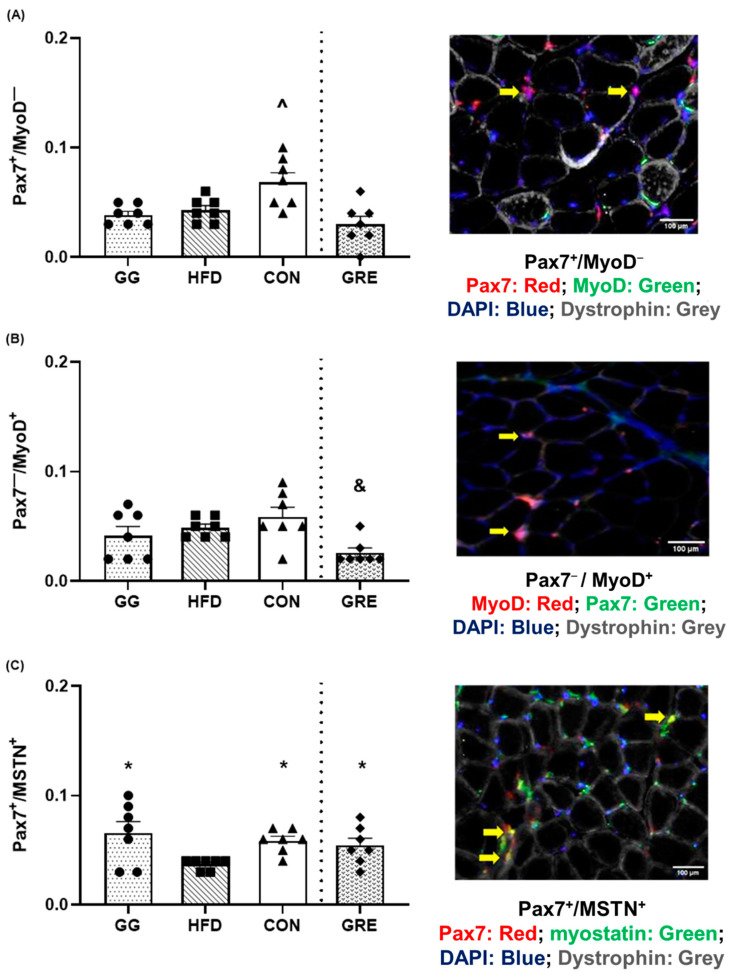
Immunohistochemical analyses and representative images for (**A**) Pax7^+^/MyoD^−^, (**B**) Pax7^−^/MyoD^+^, and (**C**) Pax7^+^/MSTN^+^. A significant condition effect was observed for Pax7^+^/MyoD^−^, Pax7^−^/MyoD^+^, and Pax7^+^/MSTN^+^. All samples were co-stained with DAPI+. Two separate one-way ANOVAs were conducted between supplementation groups (GG, HFD, and CON; HFD, CON, and GRE). Values are mean ± SE. ^ *p* < 0.05 vs. HFD, GG, GRE. & *p* < 0.05 vs. CON, HFD. * *p* < 0.05 vs. HFD.

The number of Pax7^+^/MyoD^−^ (quiescent SC) cells was greater in CON (0.064 ± 0.01 cells) than in HFD (0.039 ± 0.004 cells), GG (0.035 ± 0.004 cells), and GRE (0.029 ± 0.007 cells) (Figure 3A).

The number of Pax7^−^/MyoD^+^ (SC committed to differentiation) cells was greater in CON (0.056 ± 0.01 cells) and HFD (0.048 ± 0.003 cells) than in GRE (0.024 ± 0.004 cells) (Figure 3B).

The number of Pax7^+^/MSTN^+^ (quiescent SC) cells was greater in CON (0.057 ± 0.004 cells), GG (0.065 ± 0.01 cells), and GRE (0.055 ± 0.07 cells) than in HFD (0.034 ± 0.003 cells) (Figure 3C). No differences were observed in Pax7^+^/MyoD^+^ cells in all groups.

### 3.3. Muscle CSA

**Figure 4:**
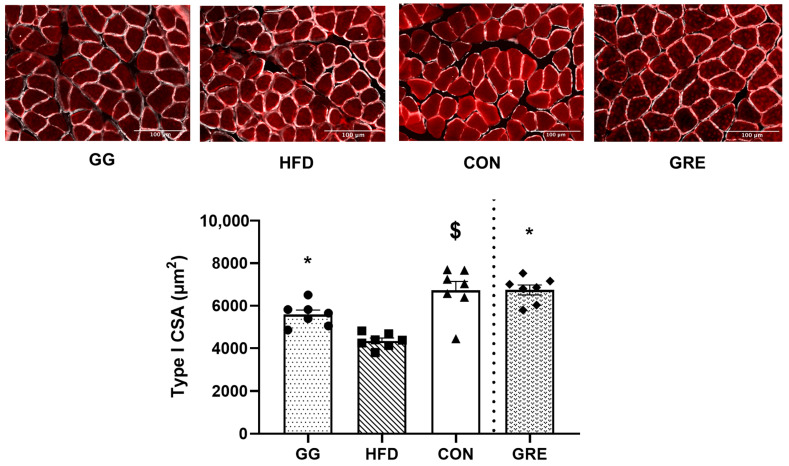
Type I CSA analyses for soleus muscle of rats. For CSA analysis [GG: *n* = 7; HFD: *n* = 7; regular diet (CON): *n* = 7; GRE: *n* = 7], around 100 muscle fibers from each rat were analyzed. A significant condition effect was observed. Two separate one-way ANOVAs were conducted between supplementation groups (GG, HFD, and CON; HFD, CON, and GRE). Values are mean ± SE. $ *p* < 0.05 vs. GG and HFD. * *p* < 0.05 vs. HFD.

GRE (6741.17 ± 232.68 μm^2^) and GG (5,584.61 ± 208.01 μm^2^) had a significantly greater CSA than HFD (4351.02 ± 127.46 μm^2^), while CON (7099.89 ± 187.33 μm^2^) was significantly greater than GG (5,584.61 ± 208.01 μm^2^) and HFD (4351.02 ± 127.46 μm^2^) (Figure 4). No differences were observed among all the other groups.

## 4. Discussion

The major finding of this study is that T2D rats (HFD) had a smaller CSA, less intramuscular *Pax7* and *MyoD* gene expression, myostatin and MyoD protein expression, and a lower number of quiescent SC (Pax7^+^/MyoD^−^) compared to non-T2D rats (CON) in the soleus muscles. When T2D rats were supplemented with GGOH (GG) or ginger (GRE) alone, they had greater muscle CSA, mitigated reductions in MyoD protein and quiescent SC number (Pax7^+^/MSTN^+^), compared to T2D rats (HFD). Additionally, when T2D rats were supplemented with ginger, myostatin protein was greater, and the number of SCs committed to differentiation (Pax7^−^/MyoD^+^) was lower than in HFD and CON.

The diabetic environment characterized by pro-inflammatory conditions and excessive oxidative stress within the skeletal muscle has been shown to reduce the SC pool and impair myogenesis [17,18,19,27,28], leading to a decrease in muscle mass [27]. Thus, identifying means to alleviate ROS and inflammation could be essential in improving SC function and muscle mass. To address this issue, our study investigated the potential benefits of two supplements, GGOH and ginger, both known for their antioxidative and anti-inflammatory properties. GGOH has been demonstrated to reduce the expression of nuclear factor-κB (NF-κB) [41], suppress LPS-induced increase in interleukin-1β (*IL-1β*), tumor necrosis factor-alpha (*TNF-α*), *IL-6*, and cyclooxygenase-2 (*COX-2*) [42], increase the synthesis of ubiquinone [43], and suppress generation of superoxide anions [44]. Similarly, ginger compounds ([6]-gingerol, [8]-gingerol, [10]-gingerol, and [6]-shogaol) have been shown to scavenge superoxide radicals [45,46] and hydroxyl radicals [46], reduce the concentrations of malondialdehyde [47,48], and decrease high-sensitivity C-reactive protein, TNF-α, and IL-6 [49]. Furthermore, based on our previous research, GGOH [30] and ginger [31] in T2D rats reduced inflammation by preventing mitochondrial fragmentation and improved clearance of damaged organelles [30] and ROS by decreasing H_2_O_2_ concentration and increasing SOD2 mRNA [31], respectively, which may have helped preserve muscle CSA. However, it remains unclear whether this preservation of muscle mass is related to the basal myogenic state of SCs, and thus, this study aimed to explore this further. The abundance of SCs, or the size of the SC pool in its quiescent state, is critical for maintaining muscle mass [16]. However, it has been suggested that the diabetic environment negatively affects the size of the SC pool [50].

Our results have demonstrated that the number of quiescent SCs (Pax7^+^/MyoD^−^) was greater in CON than in HFD, either with or without supplementation (GG and GRE). Despite the fact that the number of Pax7^+^/MyoD^−^ cells was not different, the number of Pax7^+^/MSTN^+^ quiescent cells was higher in those receiving GG and GRE compared to those that did not (HFD) and was comparable to the control condition (CON). It has been suggested that the presence of myostatin helps maintain SC quiescence and prevents SC activation [22]. Our results were aligned with a previous in vitro study demonstrating that when SCs incubated in a hyperglycemic condition (i.e., high-glucose medium), the number of SCs in a quiescence state was lower than when SCs incubated in a control condition (i.e., low-glucose medium) [51]. Previous research has suggested that a low number of quiescent satellite cells (SCs) in an aging population could hinder myogenic progress in response to stimuli, resulting in myogenic impairment and consequently diminishing muscle health [52,53]. Although this concept has not been demonstrated in diabetic muscles, our observation aligns with this theory. Similarly, in comparison to other models, such as hind limb suspension [54], aging [55], and chronic muscle wasting [56], a reduction in the overall abundance of satellite cells is observed, accompanied by muscle atrophy [54,55,56]. In rats fed with a high-fat diet (HFD), a reduced number of quiescent SC at basal state coincided with a decrease in CSA when compared to CON, GG, and GRE. It is noteworthy to mention that none of the rats were subjected to any muscle-damaging or exercise protocols. The observed differences in the size of the satellite cell (SC) pool among groups were solely the result of the high-fat diet/STZ and the respective supplementation. Interestingly, there was a lower number of cells committed to differentiation (Pax7^−^/MyoD^+^) for GRE than CON and HFD. Although speculative, a reduction in differentiating SCs could be attributed to the decrease in oxidative stress due to ginger supplementation [31], thereby alleviating muscle degeneration signaling cascades and negating the need for SC myogenesis while maintaining CSA. Lastly, no differences in Pax7^+^/MyoD^+^ SCs (proliferation) were observed across all groups. This lack of changes may be due to the absence of significant muscle damage in the skeletal muscle of these T2D rats, as SCs typically show a robust response after muscle damage/injury [9]. In addition, the muscle sampling timepoint might not capture the change of HFD-indued myogenic state either with or without supplementation. Lastly, these fibers could have regenerated into adult muscle fibers over 8 weeks. Hence, minimal changes were observed in proliferation and differentiation states, while increases were observed in MyoD protein (also observed in healthy adult muscle fibers) [46,47,48].

MyoD is the master regulator of SC myogenesis, which is essential for SC proliferation and differentiation [57]. Our report showed that MyoD protein levels were lower in T2D rats compared to control rats, which is in agreement with previous studies demonstrating MyoD reduction in STZ-induced diabetic rodents [17,58] and C2C12 cells incubated in high-glucose medium [51]. Notably, supplementation with either GGOH and ginger in T2D rats had greater MyoD protein levels compared to HFD and was comparable to CON. A previous study demonstrated that incubation of C2C12 cells with GGOH did not alter *MYOD* transcript levels but increased myogenin and MHC protein [31]. Despite the lack of MyoD protein analysis, the increased myogenin and MHC protein could indirectly suggest an increase in MyoD protein as it initiates a set of myogenic transcription factors (e.g., myogenin and MHC) that coordinate myoblast differentiation [57]. Similarly, GRE also demonstrated increases in MyoD protein. Incubating human myoblasts with ginger has been shown to increase myotube formation and improve fusion [33], suggesting an elevation in MyoD content as it is essential for differentiating myoblasts [57]. Moreover, MyoD is expressed in SCs and can also be expressed in adult muscle fibers [46,47,48], which may account for the increase in MyoD protein content observed in our study, with no changes observed in Pax7^+^/MyoD^+^ SC at the basal levels. The rise in MyoD protein with GGOH and ginger could indicate adult muscle fibers due to a reduction in inflammation [30] and ROS [31], which prevents muscle degradation pathways, hence possibly explaining the preservation of skeletal muscle mass observed in this study.

Lastly, myostatin is a negative regulator of muscle growth [12,59,60]. We have observed a lower intramuscular myostatin in the T2D rats (HFD) than CON, which were aligned with others who have reported a lower myostatin in mice injected with STZ [58] and in T2D patients [25]. Han et al. further hypothesized that the ‘accelerator–brake’ model [61] might explain the systemic myostatin decrease in T2D [25]. In T2D, insulin resistance prevents muscle growth (accelerator function); thus, myostatin (i.e., the brake for muscle growth) might reduce as there is no need for a brake as there is not an increased accelerator function [25]. Skeletal muscle may attempt to counteract muscular atrophy and hyperglycemia by decreasing intramuscular myostatin [58]. This model could, at least partly, explain the decrease in myostatin observed in the T2D group (with a reduction in CSA) in our study. Furthermore, the increases in myostatin protein in the CON, GG, and GRE groups are consistent with the increases observed in SCs stained for Pax7^+^/MSTN^+^.

One limitation of this study is the relatively short duration of 8 weeks, which may not be sufficient to fully capture the long-term effects of GGOH and ginger supplementation on skeletal muscle. However, 8 weeks is adequate to induce diabetes in rats, as demonstrated by previous research [62], and this period was sufficient to mitigate muscle mass loss with GGOH and ginger, as demonstrated in the current study. Moreover, while this study examined the effects of GGOH and ginger on the myogenic state of SC and muscle mass, it did not include a comparison with standard anti-diabetic treatments, leaving a gap in understanding how these supplements perform relative to conventional therapies. Future research should investigate whether GGOH and ginger supplementation are as effective as established anti-diabetic drugs to better assess their therapeutic potential. Additionally, further studies should explore the effects of these supplements on the satellite cell response following muscle damage.

## 5. Conclusions

In conclusion, the findings from the current study indicate that the supplementation of GGOH and ginger to T2D rats may have an integral role in mitigating muscle mass loss, which could be at least partly attributed to the increase in MyoD protein with no change in MyoD^+^ SC. Additionally, even though there were no differences in SC myogenic state (proliferative and differentiation) among groups, GGOH and ginger appeared to mitigate the reduction in the quiescent SC pool (Pax7^+^/MSTN^+^) observed in HFD. Given the importance of quiescent SC pool on retaining myogenic potential, along with reports that GGOH [30,41,42,43] and GRE [31,45,46,47,48] reduce inflammation and ROS, supplementing GGOH and ginger to T2D rats could improve muscle health.

## Data Availability

The raw data supporting the conclusions of this article will be made available by the authors on request.

## Supplementary Materials

The following supporting information can be downloaded at: https://www.mdpi.com/article/10.3390/cimb46110730/s1.
