# Supplementary material for: The Effect of Geranylgeraniol and Ginger on Satellite Cells Myogenic State in Type 2 Diabetic Rats
Source: Curr Issues Mol Biol. 2024 Oct 31;46(11):12299–310. doi: 10.3390/cimb46110730 (PMC11592527; doi:10.3390/cimb46110730)
Supplement: Supplementary file 1 [file cimb-46-00730-s001.zip › cimb-3268098-supplementary.pdf]

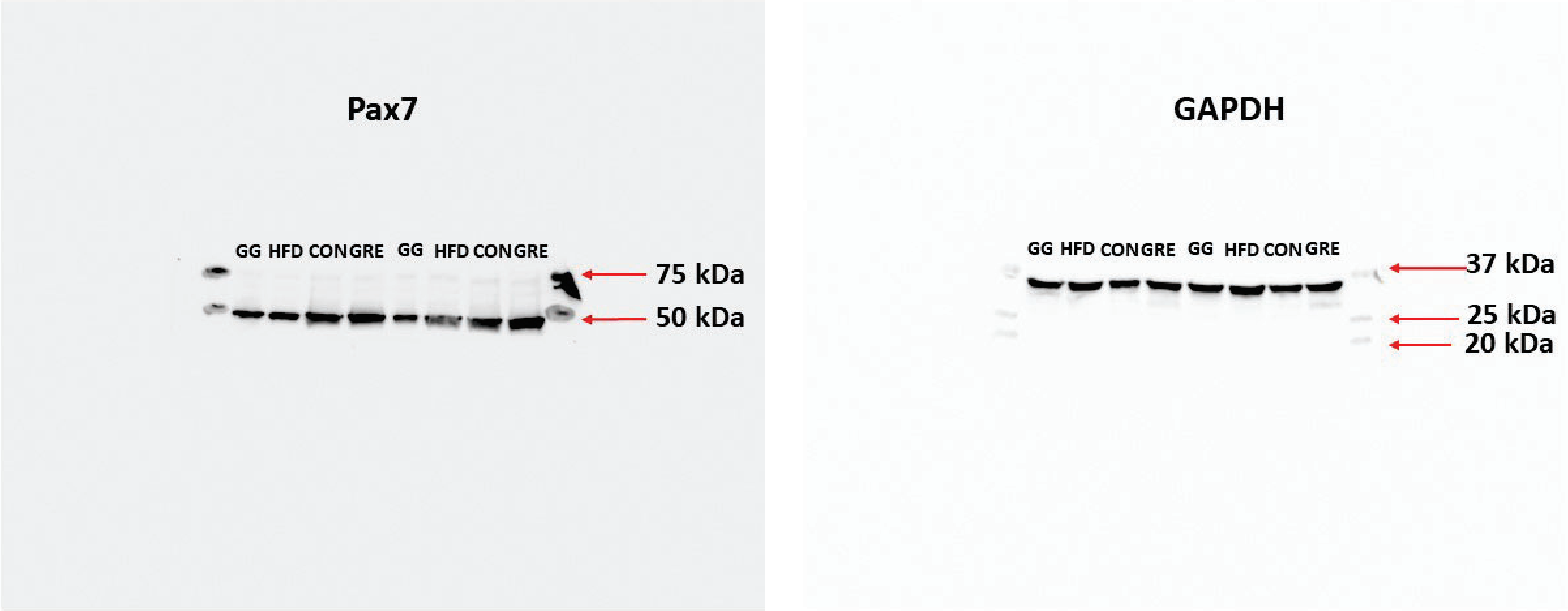

Supplemental Figure S1. Representative Full Western Blot Images for Paired box 7 (Pax7) and Glyceraldehyde-3-phosphate dehydrogenase (GAPDH; housekeeping protein). CON: control diet; HFD: high-fat diet, GG: geranylgeraniol+high-fat diet; GRE: ginger root extract+high-fat diet; kDa: kilodalton.

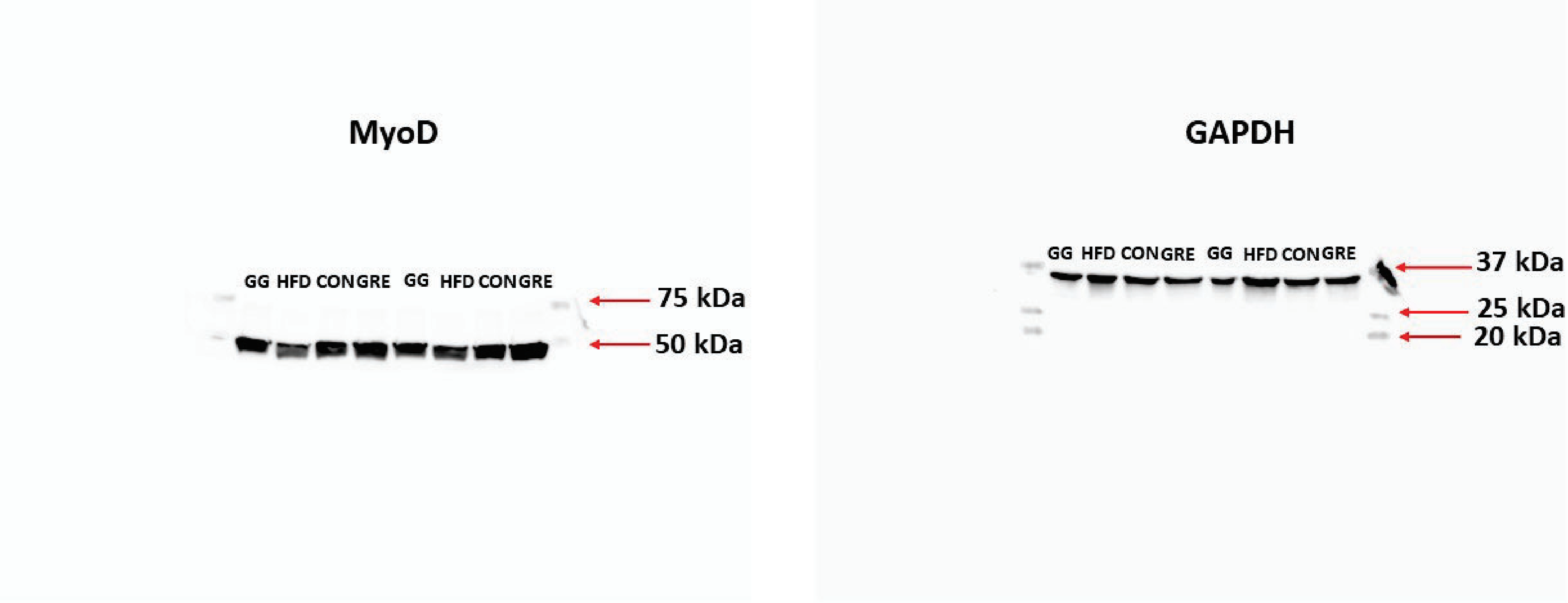

Supplemental Figure S2. Representative Full Western Blot Images for myogenic differentiation 1(MyoD) and Glyceraldehyde-3-phosphate dehydrogenase (GAPDH; housekeeping protein). CON: control diet; HFD: high-fat diet, GG: geranylgeraniol+high-fat diet; GRE: ginger root extract+high-fat diet; kDa: kilodalton.

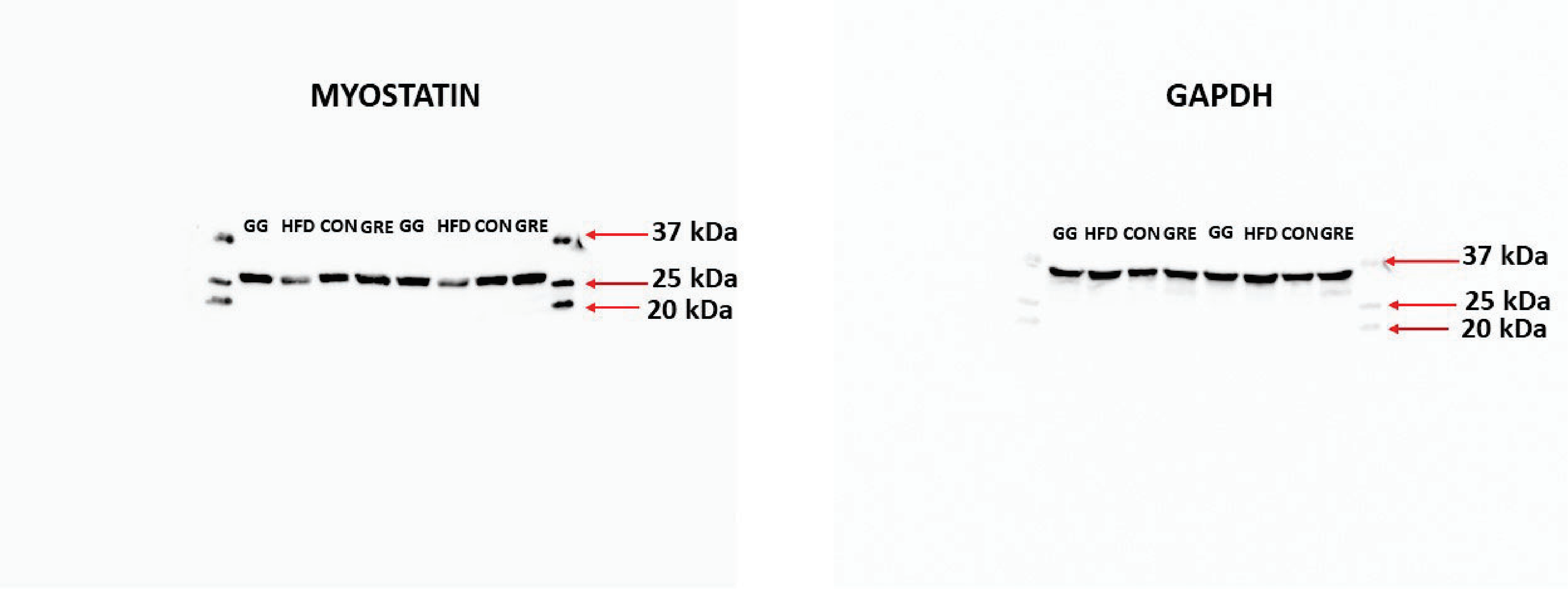

Supplemental Figure S3. Representative Full Western Blot Images for myostatin and Glyceraldehyde-3-phosphate dehydrogenase (GAPDH; housekeeping protein). CON: control diet; HFD: high-fat diet, GG: geranylgeraniol+high-fat diet; GRE: ginger root extract+high-fat diet; kDa: kilodalton.
